# Characterization of disease burden, treatment and comorbidities in a large, real-world cohort of patients with atopic dermatitis: The CorEvitas Atopic Dermatitis Registry

**DOI:** 10.1016/j.jdin.2023.11.015

**Published:** 2024-02-12

**Authors:** Jonathan I. Silverberg, Angel Cronin, Eric A. Jones, Swapna S. Dave, Robert R. McLean, Jeffrey Greenberg, Bruce Strober, Thomas Bieber, Melinda Gooderham, Amy S. Paller, Eric L. Simpson

**Affiliations:** a Department of Dermatology, George Washington University School of Medicine and Health Sciences, Washington, District of Columbia; b CorEvitas, LLC, Waltham, Massachusetts; c Department of Dermatology, Yale University School of Medicine, New Haven, Connecticut; d Central Connecticut Dermatology, Cromwell, Connecticut; e Department of Dermatology and Allergology, University Hospital of Bonn, Bonn, Germany; f Christine Kühne – Center for Allergy Research and Education, Davos, Switzerland; g SKiN Centre for Dermatology and Probity Medical Research, Peterborough, Canada; h Queen’s University, Kingston, Canada; i Departments of Dermatology and Pediatrics, Northwestern University Feinberg School of Medicine, Chicago, Illinois; j Department of Dermatology, Oregon Health & Science University, Portland, Oregon

**Keywords:** atopic dermatitis, biologic therapy, biologics, outcomes, QoL, quality of life, small molecule therapy, treatment response

*To the Editor:* Despite recent advances in systemic treatment of moderate-to-severe atopic dermatitis (AD), many eligible patients remain on topicals with inadequate control of disease.^1^ There is a growing need to assess the long-term safety and effectiveness of AD treatments in a real-world setting.^2^ The CorEvitas AD Registry was created with the objective of studying the comparative safety, effectiveness, and health outcomes of Food and Drug Administration-approved AD biologic and oral systemic treatments and off-label systemic treatments. In this study, we describe the demographic, clinical, and treatment characteristics among CorEvitas AD Registry patients in the first 2.5 years after inception and stratified by current systemic therapy use (see Supplementary Protocol for registry details, available via Mendeley at https://data.mendeley.com/datasets/d52dppkkxs/1).

From July 2020 through December 2022, 2603 patients were enrolled across 68 sites (29 US states, 5 Canadian provinces; ∼94% private practices, ∼6% academic sites), of which 43% started systemic therapy ≤12 months before enrollment, 49% started or were prescribed systemic therapy at enrollment, and 8% met the minimum disease criteria for enrollment but had no systemic therapy use at enrollment. Mean (standard deviation [SD]) age was 49.2 (18.5) years and the majority of patients were of female sex at birth (58%) and identified as White race (67%) (Table I). Among patients using or prescribed systemic therapy at enrollment, the most prescribed systemic therapy was dupilumab (80%), followed by upadacitinib (9%), and tralokinumab-ldrm (6%) (Supplementary Table I, available via Mendeley at https://data.mendeley.com/datasets/d52dppkkxs/1 and Table II).

**Table I:** Sociodemographic characteristics at enrollment in the CorEvitas Atopic Dermatitis Registry

Characteristics	Total
	*N* = 2603
Age (y)	*N* = 2602
Mean (SD)	49.2 (18.5)
Sex at birth, *n* (%)	*N* = 2601
Female	1519 (58.4%)
Race, *n* (%)	*N* = 2599
White	1736 (66.8%)
Black	341 (13.1%)
Asian	297 (11.4%)
Other∗	225 (8.7%)
Hispanic ethnicity, *n* (%)	*N* = 2593
Hispanic or Latino	208 (8.0%)
Health insurance type,†*n* (%)	*N* = 2603
Private/commercial	1663 (63.9%)
Medicare	496 (19.1%)
Medicaid	358 (13.8%)
VA/military	79 (3.0%)
Uninsured	43 (1.7%)
Education, *n* (%)	*N* = 2600
High school graduate/GED or less	816 (31.4%)
Some college, vocational training, or associate degree	911 (35.0%)
College graduate or higher	873 (33.6%)
Work status, *n* (%)	*N* = 2602
Full time	1185 (45.5%)
Part time	231 (8.9%)
Student	154 (5.9%)
Disabled	243 (9.3%)
Retired	527 (20.3%)
Stay-at-home parent/spouse	92 (3.5%)
Unemployed	170 (6.5%)
Geographic region of site, *n* (%)	*N* = 2603
Northeast	445 (17.1%)
Midwest	757 (29.1%)
South	821 (31.5%)
West	371 (14.3%)
Canada	209 (8.0%)

*GED*, General Educational Development test; *SD*, standard deviation; *VA*, US Department of Veterans Affairs.

∗ Other race includes patients who selected multiple races, American Indian or Alaskan Native, Native Hawaiian or Other Pacific Islander, or “Other race.”

† Not mutually exclusive.

**Table II:** Atopic dermatitis treatment characteristics at enrollment in the CorEvitas Atopic Dermatitis Registry

Characteristics	Total
	*N* = 2603
Current systemic treatment at enrollment,∗*n* (%)	*N* = 2603
Dupilumab	1914 (73.5%)
Upadacitinib	218 (8.4%)
Tralokinumab-ldrm	147 (5.6%)
Methotrexate	101 (3.9%)
Cyclosporine	21 (0.8%)
Apremilast	16 (0.6%)
Abrocitinib	13 (0.5%)
Mycophenolate mofetil/mycophenolic acid	8 (0.3%)
Omalizumab	5 (0.2%)
Tofacitinib	2 (0.1%)
Tacrolimus	1 (0.0%)
Other treatment at enrollment,†*n* (%)	*N* = 2603
Corticosteroids	83 (3.2%)
Phototherapy	62 (2.4%)
Topical therapy	1875 (72.0%)
History of treatment at enrollment,‡*n* (%)	*N* = 2603
Systemic therapy§	1229 (47.2%)
Corticosteroids	498 (19.1%)
Phototherapy	326 (12.5%)
Superpotent topical steroids, topical calcineurin inhibitors, or crisaborole	2257 (86.7%)
Number of systemic therapies§ before enrollment (excluding current therapy), *n* (%)	*N* = 2603
0	2356 (90.5%)
1	199 (7.6%)
2+	48 (1.8%)

∗ Frequencies may not sum to total as patients may be treated with multiple systemic therapies concomitantly.

† Not mutually exclusive. Includes both treatment in use and treatment newly started/prescribed at enrollment.

‡ Not mutually exclusive. Includes treatment started any time before enrollment, regardless of whether treatment is in use at enrollment.

§ Systemic therapy includes registry eligible biologics (tralokinumab-ldrm, dupilumab, secukinumab, ustekinumab, risankizumab-rzaa, ixekizumab, and omalizumab), eligible small molecules (abrocitinib, upadacitnib, baricitinib, apremilast, and tofacitinib), and eligible nonbiologic systemics (azathioprine, cyclosporine, methotrexate, mycophenolate mofetil, mycophenolic acid, and tacrolimus).

At enrollment, 64% of patients noted moderate-to-severe disease (vIGA-AD ≥3), mean body surface area (BSA) involvement was 20% (SD: 20%), and mean Eczema Area and Severity Index (EASI) was 11.2 (SD: 11.6). Mean Dermatology Life Quality Index (DLQI) was 8.4 (SD: 7.12), mean Patient-Oriented Eczema Measure (POEM) score was 13.3 (SD: 8.3), and 64% of patients noted Atopic Dermatitis Control Tool scores corresponding to “not controlled” AD disease (see Supplementary Tables I-V, available via Mendeley at https://data.mendeley.com/datasets/d52dppkkxs/1).

Geographic location of study site was the sociodemographic characteristic that had the largest observed differences among the groups defined by systemic therapy use (range: 82% of patients in the West started systemic therapy ≤12 months before or at enrollment [West] to 98% [Northeast]).

As expected, mean EASI scores and the percentage of patients with “not controlled” AD according to the Atopic Dermatitis Control Tool were lower among patients already using a systemic therapy compared with patients newly prescribed or not prescribed a systemic therapy. Similar patterns were observed for AD sites of involvement.

Disease-based registry cohorts offer advantages over traditional clinical trials by representing a population having exposure to different therapeutics routinely used in clinical practice, long disease duration, and notable comorbidity burden.^3^^,^^4^ Further, the registry contains clinical data (eg, disease activity scores and patient-reported outcomes) and treatment data (eg, reasons for discontinuations) that are often unavailable in claims data.^5^ However, participation in dermatology provider-based observational registries is voluntary and patient inclusion is prone to selection bias.

These real-world data can help examine comparative safety and effectiveness of various therapies, including for patients on multiple recently approved biologics and small molecules. The detailed clinical phenotypes will allow for analyses of uncommon patient subsets and understanding of unmet needs and value of various novel AD treatments.

## Conflicts of interest

Dr Silverberg has received honoraria as a consultant and/or advisory board member for AbbVie, Alamar, Aldena, Amgen, AOBiome, Arcutis, Arena, Asana, Aslan, BiomX, Biosion, Bodewell, Boehringer Ingelheim, Bristol Myers Squibb, Cara, Castle Biosciences, Celgene, Connect BioPharma, Dermavant, Dermira, DermTech, Eli Lilly, Galderma, GlaxoSmithKline, Incyte, Kiniksa, Leo Pharma, Menlo, Novartis, Optum, Pfizer, RAPT, Recludix, Regeneron, Sanofi-Genzyme, Shaperon, Union, and UpToDate; has served as a speaker for AbbVie, Eli Lilly, Leo Pharma, Pfizer, Regeneron, and Sanofi-Genzyme; and has received grants (paid to the institution) from Galderma, 10.13039/100017655Incyte, and 10.13039/100004319Pfizer. Author Cronin and Drs Jones and Dave were employees of CorEvitas, LLC at the time of this work. Dr McLean is an employee of CorEvitas, LLC. Dr Greenberg is an employee of CorEvitas, LLC. Dr Strober has received honoraria as a consultant for AbbVie, Almirall, Amgen, Arcutis, Arena, Aristea, Asana, Boehringer Ingelheim, Bristol Myers Squibb, Connect BioPharma, Dermavant, Eli Lilly, Equillium, GlaxoSmithKline, Immunic Therapeutics, Janssen, Leo Pharma, Maruho, Meiji Seika Pharma, Mindera Health, Novartis, Ortho Dermatologics, Pfizer, Regeneron, Sanofi-Genzyme, Sun Pharma, UCB, Ventyxbio, and vTv Therapeutics; has served as a speaker for AbbVie, Eli Lilly, Janssen, and Sanofi-Genzyme; coscientific director (consulting fee) for CorEvitas’ (Corrona) Psoriasis Registry; and investigator for AbbVie, Cara, CorEvitas’ (Corrona) Psoriasis Registry, Dermavant, Dermira, and Novartis. Dr Bieber has served as a speaker and/or consultant, and/or investigator for AbbVie, Affibody, Almirall, Amagma, AnaptysBio, AOBiome, Arena, Aristea, Asana Biosciences, ASLAN pharma, Bayer Health, BioVersys, Boehringer Ingelheim, Bristol Myers Squibb, Connect Pharma, Daiichi Sankyo, Dermavant, DICE Therapeutics, Domain Therapeutics, DS Pharma, EQRx, Galderma, Galapagos, Glenmark, GSK, Incyte, Innovaderm, IQVIA, Janssen, Kirin, Kymab, LEO, LG Chem, Lilly, L’Oréal, MSD, Medac, Novartis, Numab, OM-Pharma, Pfizer, Pierre Fabre, Q32 Bio, RAPT, Sanofi/Regeneron, UCB, and Union Therapeutics; and is the founder and chairman of the board of the nonprofit biotech “Davos Biosciences.” Dr Gooderham has served as an investigator, speaker, and/or adviser for AbbVie, Amgen, Akros, Arcutis, Aristea, AnaptysBio, Apogee, Bausch Health, BMS, Boehringer Ingelheim, Celgene, Dermira, Dermavant, Eli Lilly, Galderma, GSK, Incyte, Janssen, Kyowa Kirin, LEO Pharma, MedImmune, Meiji, Merck, MoonLake, Nimbus, Novartis, Pfizer, Regeneron, Roche, Sanofi-Genzyme, Sun Pharma, Tarsus, Takeda, UCB, Union, and Ventyx. Dr Paller has served as an investigator for AbbVie, Dermavant, Eli Lilly, Incyte, Janssen, Krystal, and UCB; has served as a consultant for Aegerion Pharma, Azitra, BioCryst, Boehringer Ingelheim, Bristol Myers Squibb, Castle Creek, Eli Lilly, Janssen, Krystal, LEO Pharma, Novartis, Regeneron, Sanofi/Genzyme, Seanergy, TWI Biotechnology, and UCB; and as a data safety monitoring board member for AbbVie, Abeona, Catawba, Galderma, and InMed. Dr Simpson has received consulting fees from AbbVie, Amgen, Arena Pharmaceuticals, Aslan Pharma, Benevolent AI Bio Limited “BAI,” BiomX Ltd, Bluefin Biomedicine Inc, Boehringer Ingelheim, Boston Consulting Group, Collective Acumen, LLC (CA), Coronado, Dermira, Eli Lilly, Evidera, Excerpta Medica, Galderma, GlaxoSmithKline, Forte Bio RX, Incyte Dermatologics, Janssen, Kyowa Kirin Pharmaceutical Development, Leo Pharm, Medscape LLC, Merck, Novartis, Ortho Galderma, Pfizer, Physicians World LLC, Pierre Fabre Dermo Cosmetique, Regeneron, Roivant, Sanofi-Genzyme, SPARC India, Trevi Therapeutics, WebMD, and Valeant; has served as a speaker for AbbVie, Leo, Eli Lilly, Medscape, Pfizer, Regeneron, Sanofi-Genzyme; has received advisory or steering committee fee from Arena Pharmaceuticals, Eli Lilly, GlaxoSmithKline, Incyte, Janssen, Kyowa Kirin Pharmaceutical Development, Leo, Pfizer, Regeneron and Sanofi-Genzyme; has received grants or contracts from AbbVie, Amgen, Arcutis, Aslan, Celgene, CorEvitas, Dermavant, Dermira, Eli Lilly, Galderma, Incyte, Kymab, Kyowa Hakko Kirin, Leo Pharmaceuticals, Merck, Novartis, Pfizer, Regeneron, Sanofi, and TARGET-DERM; and has been a PI for CorEvitas (paid to the institution).
